# Investigating attitudes and framing moral responsibility in healthcare professionals for smoking cessation interventions

**DOI:** 10.1111/bjhp.70025

**Published:** 2025-10-02

**Authors:** Angela Difeng Wu, Jamie Hartmann‐Boyce, Rafael Perera, Rachna Begh, Nicola Lindson

**Affiliations:** ^1^ Nuffield Department of Primary Care Health Sciences University of Oxford Oxford UK; ^2^ Department of Health Promotion and Policy University of Massachusetts Amherst Massachusetts USA

**Keywords:** behaviour change, framing, healthcare professionals, responsibility, smoking, smoking cessation

## Abstract

**Objectives:**

Evidence‐based support from healthcare professionals improves smoking cessation outcomes, yet intervention rates among UK general practitioners (GPs) remain suboptimal. This exploratory study explored whether framing messages around moral responsibility influences clinicians' intentions to offer smoking cessation support and explored their attitudes towards smoking.

**Design:**

A between‐subjects online experiment was conducted in May 2023 with 300 UK‐based GPs and medical students.

**Methods:**

Participants were randomised to one of three message conditions: professional obligation, shared responsibility, or neutral control. They rated their desire, duty, and intention to offer cessation support across clinical scenarios and completed attitude measures.

**Results:**

Compared with control, professional obligation framing was associated with higher intention scores (*β* = .20, 95% CI [.01, .39]); shared responsibility showed no effect. Subgroup analyses suggested stronger effects among medical students. Contextual factors were influential: higher scores were observed for cardiovascular disease (*β* = .80) and bipolar disorder (*β* = .21), while time pressure and patient disinterest reduced intention (*β* = −.15 and −.14). Attitudes were mixed: 70% viewed smoking as a lifestyle choice, while 88% agreed addiction is a disease.

**Conclusions:**

Professional obligation framing was associated with clinicians' intentions to offer cessation support, particularly among early‐career clinicians. Attitudinal inconsistencies highlight a disconnect between clinicians' perceptions and public health guidance. Responsibility‐based messaging may be promising for education and training. Given single‐item outcomes and the exploratory design, findings should be interpreted cautiously and future work should examine measurement properties more rigorously.

**Statement of contribution**

**What is already known on this subject?**

- Evidence‐based cessation support from clinicians improves outcomes, but delivery in UK practice is low.
- Clinicians' attitudes and sense of responsibility influence whether cessation advice is provided.
- Framing can shape health intentions, but little is known about its influence of responsibility framing on clinicians' behaviours.

**What does this study add?**

- Professional obligation framing was associated with higher intentions to support cessation.
- Associations appeared stronger among medical students, suggesting educational relevance.
- Attitudes showed inconsistencies: smoking viewed both as lifestyle choice and as disease.

## INTRODUCTION

In the UK, general practitioners (GPs) receive financial incentives to document smoking status and provide cessation advice or treatment, especially for patients with conditions such as coronary heart disease (CHD) and serious mental illnesses. Despite these incentives, intervention rates remain relatively low, with clinicians more frequently providing educational advice (Wu et al., 2025) rather than comprehensive evidence‐based pharmacological (Lindson et al., 2023; Thomas et al., 2021) or behavioural support (Hartmann‐Boyce et al., 2021).

### Healthcare professional attitudes towards smoking

The Office for Health Improvements and Disparities in the UK has emphasized that smoking is not merely a lifestyle choice but a dependency necessitating treatment (Office for Health Improvement and Disparities, 2022). Despite acknowledging this, physicians often see patients' continued smoking as a choice (Wakefield et al., 2003) and perceive numerous barriers to offering smoking cessation treatment to patients (Conlon et al., 2017), with perceptions of patient motivation and personal responsibility serving as prominent deterrents (van Eerd et al., 2017). A qualitative study focusing on physicians' views of supporting smoking cessation in patients with chronic obstructive pulmonary disease (COPD) found that physicians viewed patients who continued to smoke negatively (van Eerd et al., 2017). Additionally, recent evidence suggests that smoking‐related stigma in healthcare settings can deter help‐seeking behaviour and influence the care patients receive, further complicating provider–patient interactions (Draucker et al., 2020).

Clinician views included feeling that smoking patients did not take responsibility for their health and were somehow failing and letting their clinicians down. This view ignores the genetic and environmental factors influencing smoking behaviour (Sullivan & Kendler, 1999) and reflects societal norms blaming individuals for health issues (Wakefield et al., 2003). Studies also reveal insufficient training and low prioritization of smoking cessation relative to primary health conditions as barriers to providing support among healthcare professionals (Luxton et al., 2019; Meijer et al., 2018; Sheals et al., 2016). The idea that smoking is a personal choice is at odds with the addictive nature of cigarettes. However, a clinician can simultaneously hold the idea that cigarettes are addictive and that patients require medical support to stop using them, while also viewing their patients as actively choosing to damage their health.

### Framing theory and behaviour change

Framing theory, which examines how information presentation affects attitudes and behaviours, has potential for changing health behaviours like smoking cessation (Gong et al., 2013; Lecheler & de Vreese, 2011; McDonald et al., 2021). Framing effects demonstrate how opinions can change based on how information is presented (Kahneman & Tversky, 1982). For example, people may respond differently to factually equivalent messages depending on whether they are framed to focus on the benefits (gain‐framed) or the costs (loss‐framed) (Arendt et al., 2018; Toll et al., 2008).

### Moral responsibility

Past research has explored the impact of responsibility on behaviour change (Hallahan, 1999; Iyengar & Lenart, 1989; Jessop et al., 2008; Temmann et al., 2021), but there is a gap in understanding how framing moral responsibility influences healthcare providers' intentions. To hold an individual morally responsible for their action, they need to fulfil two conditions for moral responsibility: (Fischer & Ravizza, 1998) the individual must have sufficient awareness of the likely consequences of their actions, including understanding their moral significance, and have adequate control of their actions (Brown & Savulescu, 2019). The nuanced relationship between cigarette smoking and free will, or the ability to have adequate control over actions, is beyond the scope of this article (see) (Baumeister, 2017); however, it is essential to consider that self‐responsibility may influence how clinicians interact with patients who smoke.

Given the moral responsibility of healthcare providers, it is crucial to examine how their perceptions of smoking cessation influence their clinical practice. The concept of self‐responsibility may shape these perceptions, potentially affecting the level of support offered to patients who smoke. For instance, if a clinician perceives smoking cessation as primarily the patient's responsibility, they may be less likely to offer comprehensive support or use evidence‐based treatments. On the other hand, clinicians who view smoking cessation as a shared responsibility may be more proactive in offering support and using effective treatments. Through taking the Hippocratic oath (Oxtoby, 2016), doctors express their intention to treat patients to the best of their ability. However, the rates of evidence‐based smoking cessation treatments in UK general practice remain low despite the introduction of financial incentives (Szatkowski & Aveyard, 2016; Wu et al., 2025). This raises questions about the factors contributing to this gap between policy guidance and GP behaviour and how they can be addressed to ensure that all patients receive optimal support for smoking cessation.

*Professional obligation* refers to the ethical and professional duty that healthcare providers have to follow established medical guidelines and best practices (Toll et al., 2008). This obligation is externally imposed by medical standards, policies, and ethical codes, such as the General Medical Council's (GMC) expectations for UK physicians. Within this framework, doctors are expected to offer interventions that align with evidence‐based care. This includes routinely considering and offering smoking cessation support, in accordance with national and international clinical guidelines (Office for Health Improvement and Disparities, 2022).

*Shared responsibility*, on the other hand, reflects the idea that responsibility for a patient's health outcomes is co‐constructed between the patient and their healthcare provider (Brown & Savulescu, 2019). In this study, we use the term “shared responsibility” to reflect the concept of dyadic responsibility described by Brown and Savulescu (Brown & Savulescu, 2019), which emphasizes that responsibility can unfold across time and be distributed between multiple agents in a relational context. Here, the doctor is not solely accountable for behaviour change but contributes to it as part of an ongoing, interpersonal dynamic. Unlike professional obligation, which is defined by institutional mandates, shared responsibility emphasises mutual influence, shared agency, and collaborative decision‐making.

While these constructs may overlap in real‐world settings, they represent distinct theoretical perspectives on clinician behaviour. In this study, they were operationalised through different message framings. Although we did not empirically test their separability, future research could explore this using psychometric validation methods. Given that responsibility framing has been shown to influence health behaviours, this study explores how different responsibility frames (professional obligation vs. shared responsibility) impact healthcare providers' intentions to offer smoking cessation interventions.

### Research objective

While previous research has explored the general concept of responsibility and its impact on behaviour change (Davies et al., 2022; de Graaf et al., 2015; Park et al., 2020), there remains a gap in understanding how framing information, using moral responsibility as a theme, influences healthcare providers' intentions and behaviours. This study seeks to address this gap by exploring the influence of attitudes and how different responsibility frames are associated with healthcare providers' reported intentions. This research was exploratory in nature, meaning we did not have a pre‐specified hypothesis, but we anticipated that framing messages around professional obligation and shared responsibility might affect clinicians' expressed desire, sense of duty, and intention to provide smoking cessation support. Prior work distinguishing desire, duty, and intention suggests these items can behave differentially in relation to behaviour, motivating examination of each observed response independently in the present experiment (Smit et al., 2011). We also examined potential differences between GPs and medical students, as these two groups represent different stages in professional development. GPs are the primary providers of smoking cessation advice in UK general practice, while medical students are still forming professional norms and attitudes. Understanding how both groups respond to different responsibility frames can help inform both ongoing clinical practice and early‐stage medical education.

## MATERIALS AND METHODS

We conducted a between‐subjects online experiment in May 2023 with 300 UK‐based doctors and medical students, recruited via online advertisements and in‐person outreach. Participants (aged 18+) completed a 10‐minute Qualtrics (Qualtrics, 2020) survey after providing informed consent. Ethical approval was granted by the University of Oxford Medical Sciences Interdivisional Research Ethics Committee (Ref: R86099/RE00). The study was preregistered on the Open Science Framework and followed institutional survey research guidelines (CUREC. Internet‐Mediated Research, 2021).

### Design

**FIGURE 1:**
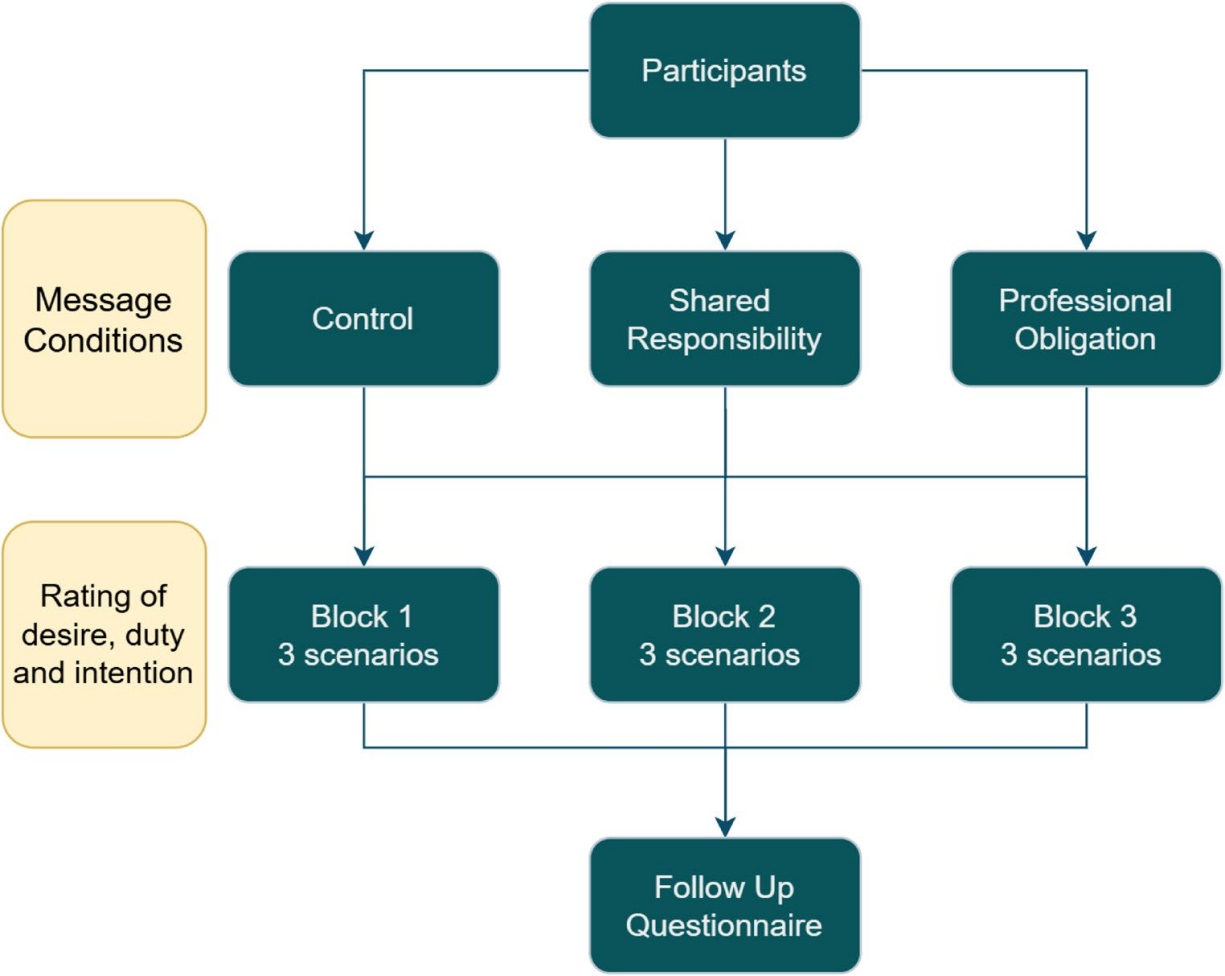
Participant flow through the experiment. Message framing was randomized between participants (3 levels). Each participant completed three clinical scenarios, systematically varied such that each illness context and each consultation context appeared once, yielding an overall 3 × 3 × 3 factorial design.

Message framing (professional obligation; shared responsibility; neutral control) was randomised between participants (3 levels). After viewing a tweet‐style message simulating an official post from a university department, participants completed three clinical scenarios. Across scenarios, illness context (cardiovascular disease; bipolar disorder; unrelated condition) and consultation context (on schedule/neutral; patient expresses no intention to quit; time pressure/behind schedule) varied within participants such that each level of illness and each level of consultation context appeared once per participant (balanced blocks across the sample). Participants then rated desire, sense of duty, and intention to intervene, along with general attitudes towards smoking (see Figure 1 for participant flow). Thus, the design comprised one between‐participants factor (framing, 3 levels) and two within‐participants factors (illness, 3 levels; consultation, 3 levels), yielding a 3 × 3 × 3 structure overall. A debrief was provided upon completion; participants were offered a £15 voucher.

### Message conditions

Each message used gain‐framed wording to reinforce that behavioural support and/or medication are effective for smoking cessation. We held message valence constant to minimise differences due to valence and focus on responsibility framing. The only variation was the second sentence (Table 1). Messages were visually designed to appear as official tweets, with style and structure consistent across conditions.

**TABLE 1:** Targeted message by frame condition.

Condition	Target message
Professional Obligation	GP failure to provide a patient with smoking cessation support is a failure to meet professional obligations
Shared Responsibility	GPs should remind patients they need not quit on their own and offer patients smoking cessation support
Control	GPs should give all patients smoking cessation support

### Scenarios

Participants viewed three scenarios drawn from a 3 (illness) × 3 (consultation) template. Scenarios were randomised and grouped into three balanced blocks so that each participant viewed three scenarios such that all illness levels and all consultation levels appeared exactly once per participant (balanced across the sample) (Table 2). Cardiovascular disease and bipolar disorder were selected as illness contexts due to their high smoking prevalence and the gap in cessation support provided to patients with these conditions (Heffner et al., 2011; Taylor et al., 2019; Wu et al., 2025; Ziedonis et al., 2008).

**TABLE 2:** Scenario builder to demonstrate the addition of the two contexts.

**Context of illness**	**+**	**Context of consultation**
Cardiovascular disease		Computer prompt
Serious mental illness		Time constraints
Non‐smoking illness‐sprained ankle		Patient previous mention of no motivation to quit

### Material testing

Five clinicians and three medical students piloted the survey and participated in follow‐up discussions. They confirmed the realism of the materials, survey clarity, and authenticity of the tweet‐style messages. Based on their feedback, we adjusted the wording in the control message, changing “GPs must give all patients smoking cessation support” to “GPs should give all patients smoking cessation support.” We also adapted our Likert scales from 7 points to 5 following the recommendations of testers.

### Measures

#### Outcome: desire, duty, and intention

In line with previous work (Smit et al., 2011), participants were asked to rate their desire, duty, and intention (Table 3) using a 5‐point scale ranging from:

1. *Strongly disagree*
2. *Somewhat disagree*
3. *Neither agree nor disagree*
4. *Somewhat agree*
5. *Strongly agree*

**TABLE 3:** Statements regarding desire, duty, and intention outcomes.

Desire	“I want to offer this patient smoking cessation support”
Duty	“I ought to offer this patient smoking cessation support”
Intention	“I intend to offer this patient smoking cessation support”

Higher scores indicate a stronger sense of desire, duty, and intention. The sequence of questions was randomised to minimise bias. Each dimension—desire, duty, and intention—was analysed separately. In this experiment, these items are treated as separate, single‐item outcomes, observed motivational responses that may differ in how they respond to the manipulation and context. We do not treat them as validated latent constructs. Analyses therefore do not compare these items to one another or claim construct separability; instead, we estimate associations between the experimental conditions/contexts and each item considered independently.

#### Attitude statements

We used a series of statements (Table 4) to assess participants' perceptions of smoking, including assertions like: ‘smoking is a lifestyle choice’ and ‘addiction is a disease that requires treatment’. Participants rated each statement on a 5‐point Likert scale, ranging from *definitely false* to *definitely true*. Items were developed by the research team to reflect themes in clinical and public‐health discourse (e.g., stigma, addiction, responsibility), drawing on commonly used UK clinical/surveillance language, and were piloted with five clinicians and three medical students for clarity and relevance. We report them transparently as study‐specific items, not validated scales.

**TABLE 4:** Health attitude statements.

Statements
It is a doctor's duty to offer help regardless of patient's own motivation
A person's health status is the result of the person's behaviours and choices
Addiction is a disease that requires treatment
Doctors can only help patients that seek help
People who engage in unhealthy lifestyles should do more to change their behaviour
Smoking is a lifestyle choice

#### Demographics

We collected anonymised demographic information, including participants' gender, ethnicity, and years of experience as a doctor or medical student in the UK.

#### Manipulation checks

Factual manipulation checks were also conducted after presenting the images to ensure that participants correctly perceived the intended message of the frames (Kane & Barabas, 2019). Participants were asked to choose the message that they felt best represented what the displayed tweet conveyed. Participants who selected a message that was not paired with the displayed tweet failed the manipulation check and therefore were excluded from a sensitivity analysis.

### Statistical analysis

Analysis was conducted in Stata 17.

#### Regression models

Primary analyses involved multiple linear regression models to estimate associations of framing conditions on healthcare professionals' reported desire, duty, and intention to offer smoking cessation support. For these models, we computed participant level mean scores for each outcome by averaging responses across the three scenarios, yielding one observation per participant. Thus, these models tested the between‐participants framing factor (3 levels), averaging across the within‐participants illness and consultation contexts. Due to baseline imbalances, these models were adjusted for age and gender. Post‐hoc sensitivity analyses without covariate adjustments were conducted and presented in Supporting Information to confirm robustness and assess outcomes under strictly randomised conditions. Additionally, pre‐specified sensitivity analyses excluding participants who failed manipulation checks (*n* = 20) were conducted. Prespecified subgroup analyses explored differences between general practitioners (GPs; *n* = 179) and medical students (*n* = 110), adjusting for age and gender.

We used mixed‐effects linear regression models to estimate associations of the within‐participants illness (3 levels) and consultation (3 levels) contexts on desire, duty, and intention, while also including framing as a between‐participants predictor. Models accounted for clustering of repeated observations within participants (random intercept for participant ID). We estimated model parameters using maximum likelihood methods and used the likelihood ratio test to assess model goodness‐of‐fit. These models were also adjusted for age and gender. We also ran prespecified subgroup analyses exploring difference between GPs and medical students.

## RESULTS

### Participants

We randomised 300 participants (Table 5) into three groups: 103 in the control group, 103 in the professional obligation group, and 94 in the shared responsibility group. Most (91.3%) had never smoked, and only 7.3% lived with someone who currently smoked. Twenty failed manipulation checks and were removed in sensitivity analyses.

**TABLE 5:** Baseline characteristics of population split by framing condition.

Characteristic	Whole cohort (*n* = 300)	Control (*n* = 103)	Professional obligation (*n* = 103)	Shared responsibility (*n* = 94)
Age				
Aged 18/24	111 (37.0%)	40 (38.8%)	36 (35.0%)	35 (37.2%)
Aged 25/34	61 (20.3%)	21 (20.4%)	21 (20.4%)	19 (20.2%)
Aged 35/44	97 (32.3%)	29 (28.2%)	39 (37.9%)	29 (30.9%)
Aged 45/54	23 (7.7%)	8 (7.8%)	5 (4.9%)	10 (10.6%)
Aged 55/64	6 (2.0%)	5 (4.9%)	1 (1.0%)	0 (.0%)
Over 65	2 (.7%)	0 (.0%)	1 (1.0%)	1 (1.1%)
Gender				
Male	100 (33.3%)	40 (38.8%)	30 (29.1%)	30 (31.9%)
Female	193 (64.3%)	59 (57.3%)	71 (68.9%)	63 (67.0%)
Non‐binary or other	5 (1.7%)	2 (1.9%)	2 (1.9%)	1 (1.1%)
Prefer not to say	2 (.7%)	2 (1.9%)	0 (.0%)	0 (.0%)
Ethnicity				
British	141 (47.0%)	45 (43.7%)	51 (49.5%)	45 (47.9%)
Irish	7 (2.3%)	2 (1.9%)	0 (.0%)	5 (5.3%)
Other White/European	19 (6.3%)	8 (7.8%)	5 (4.9%)	6 (6.4%)
White and Black Caribbean	1 (.3%)	1 (1.0%)	0 (.0%)	0 (.0%)
White and Black African	1 (.3%)	1 (1.0%)	0 (.0%)	0 (.0%)
White and Asian	11 (3.7%)	2 (1.9%)	5 (4.9%)	4 (4.3%)
Other multiple ethnic background	4 (1.3%)	0 (.0%)	1 (1.0%)	3 (3.2%)
Indian	37 (12.3%)	13 (12.6%)	14 (13.6%)	10 (10.6%)
Pakistani	17 (5.7%)	8 (7.8%)	5 (4.9%)	4 (4.3%)
Bangladeshi	10 (3.3%)	5 (4.9%)	3 (2.9%)	2 (2.1%)
Chinese	10 (3.3%)	3 (2.9%)	4 (3.9%)	3 (3.2%)
Other Asian	16 (5.3%)	5 (4.9%)	6 (5.8%)	5 (5.3%)
African	9 (3.0%)	2 (1.9%)	4 (3.9%)	3 (3.2%)
Caribbean	2 (.7%)	1 (1.0%)	1 (1.0%)	0 (.0%)
Other Black/African/Caribbean	3 (1.0%)	3 (2.9%)	0 (.0%)	0 (.0%)
Arab	6 (2.0%)	2 (1.9%)	1 (1.0%)	3 (3.2%)
Other ethnic group	6 (2.0%)	2 (1.9%)	3 (2.9%)	1 (1.1%)
Medical profession				
General practitioner GP	179 (59.7%)	59 (57.3%)	63 (61.2%)	57 (60.6%)
Medical student	110 (36.7%)	40 (38.8%)	36 (35.0%)	34 (36.2%)
Other form of medical doctor	11 (3.7%)	4 (3.9%)	4 (3.9%)	3 (3.2%)
Smoking status				
Never smoked	274 (91.3%)	93 (90.3%)	93 (90.3%)	88 (93.6%)
Used to smoke	15 (5.0%)	5 (4.9%)	5 (4.9%)	5 (5.3%)
Currently smoking	11 (3.7%)	5 (4.9%)	5 (4.9%)	1 (1.1%)
Living with a smoker	22 (7.3%)	9 (8.7%)	9 (8.7%)	4 (4.3%)
Years in medical school^a^	4.14 (.98)	4.00 (1.10)	4.11 (.90)	4.34 (.90)
Years as practicing physician^a^	13.07 (6.53)	14.33 (7.10)	12.62 (6.18)	12.30 (6.22)
Failed manipulation check	20 (6.6%)	6 (5.8%)	5 (4.9%)	9 (9.6%)

^a^ Mean and standard deviation.

### Descriptive analysis

The mean scores for desire, duty, and intention were 4.38 (SD = .61), 4.37 (SD = .66), and 4.09 (SD = .72), respectively, with higher scores indicating a more positive sense of desire, duty or intention and the maximum score being 5 (Table 6 for results stratified by experimental groups).

**TABLE 6:** Mean and standard deviation of outcomes split by message conditions.

Frame	Desire		Duty		Intention	
	Mean	SD	Mean	SD	Mean	SD
Control group	4.31	.64	4.35	.62	4.00	.75
Professional obligation	4.41	.60	4.39	.66	4.16	.71
Shared responsibility	4.42	.60	4.37	.70	4.12	.66
Total sample	4.38	.61	4.37	.66	4.09	.71

#### Breakdown of attitude statements

**FIGURE 2:**
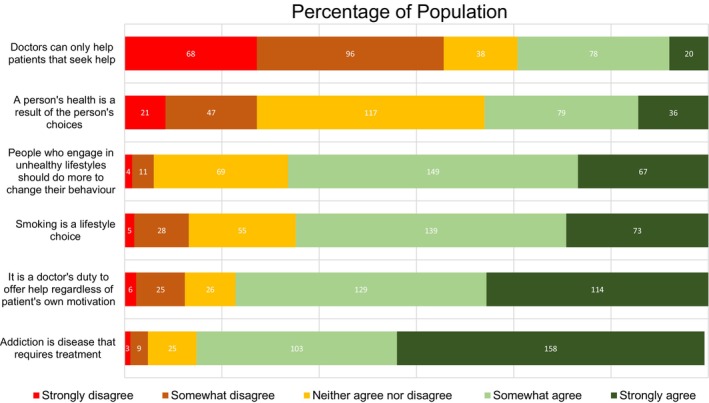
Distribution of attitudes towards health behaviours.

There was strong agreement among participants that addiction is a disease requiring treatment, with responses clustered in the ‘probably true’ (35%) and ‘definitely true’ (53%) categories. Similarly, 72% of participants agreed that individuals engaging in unhealthy lifestyles should take greater steps to change their behaviour. The statement that smoking is a lifestyle choice received substantial agreement, with 70% considering it true. However, opinions on whether health is solely the result of personal choices were more evenly distributed across all response categories (Figure 2).

#### Multivariable linear regression

We conducted three separate multivariable linear regression models to assess the association of framing conditions on clinicians' reported desire, sense of duty, and intention to provide smoking cessation support (Table 7). Each model adjusted for age and gender, which were not balanced across the randomised groups (see Table S1 for full outputs).

**TABLE 7:** Impact of framing on outcomes adjusting for age and gender.

	Professional obligation		Shared responsibility	
	*β*	95% CI	*β*	95% CI
Primary outcomes				
Desire	.11	−.06, .27	.11	−.06, .28
Duty	.04	−.13, .22	.03	−.16, .21
Intention	.20*	.01, .39	.15	−.04, .35

* *p* < .05.

Compared with the control condition, professional obligation framing was associated with higher intention scores to offer smoking cessation support (*β* = .20, 95% CI [.01, .39]). However, this framing showed no statistically significant association for desire or sense of duty. The shared responsibility framing also had no statistically significant effect on any of the three outcomes.

We conducted sensitivity analyses to assess the robustness of our multivariable linear regression findings. First, excluding participants who failed the manipulation checks (*n* = 20) did not change the main conclusions, as the professional obligation framing remained significantly associated with higher intentions to offer smoking cessation support (see Table S2). However, removing the adjustments for demographic covariates (age and gender) resulted in non‐significant effects for all framing conditions, although the directions of the observed relationships remained consistent with primary analyses (see Table S3).

Subgroup analyses by professional role (GP vs. medical student) revealed no statistically significant effects within either group (see Table S4). However, some differences in the direction and magnitude of effects were observed relative to the main analysis. Among medical students, the direction of effects under professional obligation framing was towards higher reported desire, duty, and intention. In contrast, among GPs, both framing conditions showed a negative direction of effect on reported duty compared to the control group. For desire and intention, the direction of effects among GPs was similar to that observed in the overall sample, although effect sizes were smaller.

#### Mixed‐effect linear regression

We next examined whether illness and consultation scenario contexts was associated with clinicians' responses, using mixed‐effects linear regression models on desire, duty, and intention (Table 8; Table S5).

**TABLE 8:** Mixed‐effect analysis on outcomes adjusting for age and gender.

	Desire		Duty		Intention	
	*β*	95% CI	*β*	95% CI	*β*	95% CI
Framing						
Professional obligation	.11	−.06, .27	.04	−.13, .21	.20*	.02, .39
Shared responsibility	.11	−.05, .28	.03	−.15, .20	.15	−.04, .34
Context of illness						
Bipolar disorder	.20*	.08, .31	.15*	.04, .26	.21*	.08, .33
Heart disease	.56*	.44, .67	.51*	.39, .62	.80*	.67, .93
Context of scenario						
No intention to quit	−.07	−.18, .04	−.08	−.19, .03	−.14*	−.27, −.02
Running late	.06	−.06, .17	.02	−.09, .13	−.15*	−.28, −.03

* *p* < .05.

The cardiovascular disease context was associated with significantly higher reported desire (*β* = .56, 95% CI [.44, .67]), duty (*β* = .51, 95% CI [.39, .62]), and intention to provide support (*β* = .80, 95% CI [.67, .92]) compared to an unrelated illness context. Similarly, the bipolar disorder context was associated with increases in desire (*β* = .20, 95% CI [.08, .31]), duty (*β* = .15, 95% CI [.04, .26]), and intention (*β* = .21, 95% CI [.08, .33]).

After adjusting for illness and scenario context, the professional obligation framing remained a significantly associated with intention (*β* = .20, 95% CI [.02, .39]), while the shared responsibility framing continued to show no significant effects.

Scenario context also was associated with differences in clinicians' intentions. Compared to a neutral computer prompt, encountering a patient who expressed no intention to quit smoking was associated with a significant reduction in intention (*β* = −.14, 95% CI [−.27, −.02]). Similarly, being behind schedule during the consultation was significantly associated with reduced clinicians' intention scores (*β* = −.15, 95% CI [−.28, −.03]).

Sensitivity analyses for the mixed‐effect linear regression models indicated that excluding demographic covariates (age and gender) resulted in non‐significant effects for framing conditions, although the overall pattern and direction of the effects remained similar to the fully adjusted models (see Table S6). The context of illness remained statistically significantly associated with clinicians' reported desire, duty, and intention across all sensitivity analyses.

Mixed‐effects subgroup analyses showed generally similar patterns between medical students and practicing doctors, with both groups giving higher ratings of desire, duty, and intention in response to illness contexts, particularly heart disease. In contrast, the associations of bipolar disorder were smaller and not statistically significant among medical students, while doctors showed significant increases across all three outcomes. Doctors also gave lower ratings of intention when scenarios included time pressure, whereas medical students' responses in these contexts were smaller and not statistically significant. A significant reduction in perceived duty was observed among students when the patient had no intention to quit. Medical students showed slightly larger associations of professional obligation framing on intention than doctors, though framing effects were not statistically significant in either group (see Table S7 for subgroup analysis results).

## DISCUSSION

### Main findings

This exploratory study found that professional obligation framing was associated with higher intention scores in adjusted models, although effects were modest and not consistently observed across all analyses. Contextual factors, particularly patient illness, such as cardiovascular disease and, to a lesser extent, bipolar disorder, linked to higher reported desire, duty, and intention to intervene. Subgroup analyses indicated that the association of professional obligation framing on intention was somewhat larger among medical students than GPs, though these differences were not statistically significant. Attitudes towards health responsibility were mixed, with strong agreement that addiction requires treatment, yet smoking was also viewed as a lifestyle choice.

### Interpretation and limitations

Participants' identifying smoking as a lifestyle choice suggests a disconnect with UK health policy guidelines that state smoking is an addiction (Office for Health Improvement and Disparities, 2022). While most participants strongly agreed that addiction requires treatment, many also considered smoking a lifestyle choice. Given that participants were not asked to explain their responses, we cannot infer a rejection of the addictive nature of smoking. One possibility is that participants view the initiation of smoking as a lifestyle choice, with addiction emerging only after continued use. Future research should explore how clinicians conceptualise addiction and lifestyle, and how these beliefs relate to clinical practice.

The belief that smoking is a lifestyle choice prompts examination of the relationship between personal beliefs about smoking and professional behaviours. If smoking is seen as a lifestyle choice, professionals might be less proactive in offering support, believing the responsibility to quit lies with the individual. The constraints of a typical 10‐minute consultation may also influence this perspective, prioritising other treatments over smoking cessation advice (Gopfert et al., 2021). As such, the importance given to smoking cessation advice relative to that given to other health‐related advice could reflect their personal beliefs about the onus of responsibility being on the patient.

However, in this study, participants still generally reported high intentions to offer smoking cessation support, with even greater intentions to offer support for patients with CVD or mental illness, compared to a non‐smoking related illness such as a sprained ankle. This raises questions about whether reported intentions reliably predict behaviour. Although intention is commonly used as a proxy for behaviour in implementation research, it does not always translate into action (Eccles et al., 2006). A systematic review of 78 studies found that intention accounted for between 25.6% and 34% of the variance in actual behaviour, highlighting the potential gap between motivation and real‐world practice (Godin et al., 2008). Factors such as time, competing demands, and confidence may mediate this gap.

In this study, professional obligation framing was associated with higher reported intentions to provide cessation support in the main adjusted model. Subgroup analyses suggested this association may have been more pronounced among medical students than GPs, although these comparisons were underpowered and should be interpreted cautiously. Differences between students and practicing doctors may reflect clinical experience, generational norms, or varying confidence in professional roles (McNair et al., 2016). For example, it is well documented that empathy tends to decline during medical training, which may shape clinical priorities and responsiveness to moral cues (Hojat et al., 2009; Neumann et al., 2011). These findings raise questions about the viability of framing‐based strategies to influence more experienced clinicians.

We did not include formal interaction terms (e.g., framing × illness context or framing × scenario) in our regression models, as this study was exploratory and not powered to detect such effects. Our goal was to describe general patterns in how clinicians responded to different framings and scenarios. Future work with a priori hypotheses and larger samples could explore these interactions more systematically to inform message tailoring. Although subgroup analyses suggested some differences in responses between GPs and students, we did not formally test interactions between professional role and framing condition. These findings are reported descriptively and should not be overinterpreted. Future research should examine whether professional role moderates message effects, especially in the context of developing targeted interventions.

This study did not use latent variable analysis to validate the distinctiveness of the desire, duty, and intention constructs. Our aim was to examine how these concepts, as presented through framing and context, were associated with participants' responses. We therefore refrain from making claims about the latent structure of desire, duty, and intention in this dataset and analyse them only as separate observed outcomes. Future studies may wish to use psychometric validation to empirically distinguish these constructs. Additionally, we did not examine clinicians' views on e‐cigarettes or vaping, or account for consultation format (e.g., in‐person vs. remote), which may also influence responses. These represent important directions for future work.

Although secondary analyses using the Theory of Planned Behaviour (TPB) (Ajzen, 2002) were initially pre‐registered, these were excluded here to streamline the manuscript. These supplementary analyses, available upon request, yielded results consistent with primary findings, supporting theoretical predictions.

## CONCLUSIONS

This study highlights a tension between UK health policy, which frames smoking as an addiction, and clinicians' perspectives, with many viewing it as a lifestyle choice. While participants expressed strong intentions to provide cessation support, especially in serious illness contexts, personal beliefs about responsibility and the constraints of clinical practice may influence how consistently this support is offered. Professional obligation framing showed some promise in enhancing intentions, particularly among medical students, but effects were modest and not consistent across analyses.

Future research should explore how clinicians' beliefs, contextual factors, and structural barriers interact to shape their delivery of cessation support. These insights can inform targeted interventions to align clinician attitudes and practices more closely with public health guidance. Educational and training initiatives may play a role in clarifying the addictive nature of smoking, strengthening professional responsibility, and addressing misconceptions that may hinder proactive clinical engagement with tobacco cessation.

## AUTHOR CONTRIBUTIONS

**Angela Difeng Wu:** Conceptualization; methodology; software; data curation; investigation; validation; formal analysis; visualization; project administration; resources; writing – original draft; writing – review and editing. **Jamie Hartmann‐Boyce:** Funding acquisition; writing – review and editing. **Rafael Perera:** Supervision; writing – review and editing. **Rachna Begh:** Writing – review and editing; supervision. **Nicola Lindson:** Writing – review and editing; supervision.

## Supporting information


Table S1.–S7.

## Data Availability

The data that support the findings of this study are openly available at http://doi.org/10.17605/OSF.IO/NEY9Q.
