# Supplementary material for: Investigating attitudes and framing moral responsibility in healthcare professionals for smoking cessation interventions
Source: Br J Health Psychol. 2025 Oct 2;30(4):e70025. doi: 10.1111/bjhp.70025 (PMC12491834; doi:10.1111/bjhp.70025)
Supplement: Supplementary file 1 — Table S1.–S7. [file BJHP-30-0-s001.docx]

**Supplementary table s1**
multivariable linear regression results (adjusted for age and gender)

Desire

| **Predictor** | **Coefficient** | **Std. Error** | **t** | **p-value** | **95% CI Lower** | **95% CI Upper** |
| --- | --- | --- | --- | --- | --- | --- |
| **Frame** |  |  |  |  |  |  |
| Professional obligation | 0.107 | 0.084 | 1.28 | 0.203 | -0.058 | 0.273 |
| Shared responsibility | 0.112 | 0.086 | 1.30 | 0.194 | -0.058 | 0.281 |
| **Age group** |  |  |  |  |  |  |
| 25–34 (ref) | — | — | — | — | — | — |
| 35–44 | -0.080 | 0.094 | -0.84 | 0.399 | -0.266 | 0.106 |
| 45–54 | -0.032 | 0.084 | -0.38 | 0.706 | -0.196 | 0.133 |
| 55–64 | 0.248 | 0.136 | 1.82 | 0.069 | -0.020 | 0.516 |
| 65+ | 0.548 | 0.252 | 2.18 | 0.030 | 0.053 | 1.044 |
| **Gender** |  |  |  |  |  |  |
| Male (ref) | — | — | — | — | — | — |
| Female | 0.282 | 0.074 | 3.81 | <0.001 | 0.137 | 0.428 |
| Non-binary / Other | -0.350 | 0.273 | -1.28 | 0.201 | -0.887 | 0.187 |
| Prefer not to say | 0.748 | 0.429 | 1.74 | 0.083 | -0.097 | 1.592 |

Duty

| **Predictor** | **Coefficient** | **Std. Error** | **t** | **p-value** | **95% CI Lower** | **95% CI Upper** |
| --- | --- | --- | --- | --- | --- | --- |
| **Frame** |  |  |  |  |  |  |
| Professional obligation | 0.041 | 0.090 | 0.46 | 0.649 | -0.137 | 0.219 |
| Shared responsibility | 0.026 | 0.092 | 0.28 | 0.777 | -0.156 | 0.208 |
| **Age group** |  |  |  |  |  |  |
| 25–34 (ref) | — | — | — | — | — | — |
| 35–44 | -0.207 | 0.101 | -2.04 | 0.042 | -0.406 | -0.008 |
| 45–54 | 0.001 | 0.090 | 0.01 | 0.992 | -0.176 | 0.178 |
| 55–64 | 0.138 | 0.146 | 0.94 | 0.346 | -0.149 | 0.425 |
| 65+ | 0.625 | 0.270 | 2.32 | 0.021 | 0.095 | 1.156 |
| **Gender** |  |  |  |  |  |  |
| Male (ref) | — | — | — | — | — | — |
| Female | 0.309 | 0.079 | 3.89 | <0.001 | 0.153 | 0.465 |
| Non-binary / Other | 0.172 | 0.292 | 0.59 | 0.557 | -0.404 | 0.747 |
| Prefer not to say | 0.845 | 0.460 | 1.84 | 0.067 | -0.060 | 1.751 |

Intention

| **Predictor** | **Coefficient** | **Std. Error** | **t** | **p-value** | **95% CI Lower** | **95% CI Upper** |
| --- | --- | --- | --- | --- | --- | --- |
| **Frame** |  |  |  |  |  |  |
| Professional obligation | 0.205 | 0.096 | 2.12 | 0.035 | 0.015 | 0.394 |
| Shared responsibility | 0.151 | 0.099 | 1.54 | 0.126 | -0.043 | 0.345 |
| **Age group** |  |  |  |  |  |  |
| 25–34 (ref) | — | — | — | — | — | — |
| 35–44 | -0.055 | 0.108 | -0.50 | 0.614 | -0.267 | 0.158 |
| 45–54 | -0.041 | 0.096 | -0.43 | 0.667 | -0.230 | 0.147 |
| 55–64 | 0.264 | 0.156 | 1.70 | 0.091 | -0.042 | 0.571 |
| 65+ | 0.943 | 0.288 | 3.28 | 0.001 | 0.377 | 1.510 |
| **Gender** |  |  |  |  |  |  |
| Male (ref) | — | — | — | — | — | — |
| Female | 0.173 | 0.085 | 2.04 | 0.042 | 0.006 | 0.339 |
| Non-binary / Other | -1.008 | 0.312 | -3.23 | 0.001 | -1.623 | -0.393 |
| Prefer not to say | 1.021 | 0.491 | 2.08 | 0.039 | 0.054 | 1.988 |

**supplementary table s2**
sensitivity analysis: excluding failed manipulation check participants

|  | **Professional obligation** | | **Shared responsibility** | |
| --- | --- | --- | --- | --- |
|  | **Β** | **95% ci** | **Β** | **95% ci** |
| **Primary outcomes** |  |  |  |  |
| Desire | 0.12 | -0.04, 0.28 | 0.08 | -0.08, 0.25 |
| Duty | 0.12 | -0.05, 0.28 | 0.04 | -0.14, 0.21 |
| Intention | 0.23* | 0.04, 0.42 | 0.11 | -0.08, 0.31 |

*p<0.05

**supplementary table s3**
unadjusted multivariable linear regression results (no covariate adjustment)

|  | **Professional obligation** | | **Shared responsibility** | |
| --- | --- | --- | --- | --- |
|  | **Β** | **95% ci** | **Β** | **95% ci** |
| **Primary outcomes** |  |  |  |  |
| Desire | 0.10 | -0.6, 0.27 | 0.11 | -0.06, 0.28 |
| Duty | 0.04 | -0.14, 0.22 | 0.02 | -0.16, 0.20 |
| Intention | 0.16 | -0.03, 0.36 | 0.13 | -0.07, 0.33 |

**supplementary table s4**
subgroup analysis: medical professional role status (adjusted for age and gender)

|  | **Professional obligation** | | **Shared responsibility** | |
| --- | --- | --- | --- | --- |
| **GP** | **Β** | **95% ci** | **Β** | **95% ci** |
| **Primary outcomes** |  |  |  |  |
| Desire | 0.14 | -0.08, 0.36 | 0.08 | -0.14, 0.31 |
| Duty | -0.07 | -0.31, 0.16 | -0.02 | -0.26, 0.21 |
| Intention | 0.18 | -0.07, 0.43 | 0.15 | -0.11, 0.41 |

|  | **Professional obligation** | | **Shared responsibility** | |
| --- | --- | --- | --- | --- |
| Medical Student | **Β** | **95% ci** | **Β** | **95% ci** |
| **Primary outcomes** |  |  |  |  |
| Desire | 0.05 | -0.18, 0.27 | 0.14 | -0.09, 0.38 |
| Duty | 0.22 | -0.03, 0.48 | 0.07 | -0.18, 0.33 |
| Intention | 0.25 | -0.02, 0.52 | 0.17 | -0.11, 0.45 |

**supplementary table s5**
mixed-effects models (adjusted for age and gender)

Desire

| Predictor | Coefficient | Std. Error | z | p-value | 95% CI Lower | 95% CI Upper |
| --- | --- | --- | --- | --- | --- | --- |
| Frame |  |  |  |  |  |  |
| Professional obligation | 0.107 | 0.083 | 1.30 | 0.194 | -0.055 | 0.269 |
| Shared responsibility | 0.112 | 0.085 | 1.32 | 0.185 | -0.054 | 0.278 |
| Context of illness |  |  |  |  |  |  |
| Bi-polar | 0.197 | 0.058 | 3.40 | 0.001 | 0.083 | 0.310 |
| Heart disease | 0.557 | 0.058 | 9.63 | 0.000 | 0.444 | 0.671 |
| Context of scenario |  |  |  |  |  |  |
| Last record stated no intention to quit | -0.071 | 0.058 | -1.22 | 0.223 | -0.184 | 0.043 |
| Running behind schedule | 0.056 | 0.058 | 0.97 | 0.334 | -0.058 | 0.169 |
| Age |  |  |  |  |  |  |
| 25–34 (ref) | — | — | — | — | — | — |
| 25–44 | -0.080 | 0.093 | -0.86 | 0.390 | -0.261 | 0.102 |
| 35–44 | -0.032 | 0.082 | -0.38 | 0.701 | -0.193 | 0.130 |
| 45–54 | 0.248 | 0.134 | 1.86 | 0.063 | -0.013 | 0.510 |
| 55–64 | 0.548 | 0.247 | 2.22 | 0.026 | 0.065 | 1.032 |
| Over 65 | 0.632 | 0.416 | 1.52 | 0.128 | -0.183 | 1.446 |
| Gender |  |  |  |  |  |  |
| Male (ref) | — | — | — | — | — | — |
| Female | 0.282 | 0.073 | 3.89 | 0.000 | 0.140 | 0.424 |
| Non-binary / Other | -0.350 | 0.268 | -1.31 | 0.191 | -0.875 | 0.175 |
| Prefer not to say | 0.748 | 0.421 | 1.77 | 0.076 | -0.078 | 1.573 |

Duty

| Predictor | Coefficient | Std. Error | z | p-value | 95% CI Lower | 95% CI Upper |
| --- | --- | --- | --- | --- | --- | --- |
| Frame |  |  |  |  |  |  |
| Professional obligation | 0.041 | 0.089 | 0.46 | 0.643 | -0.133 | 0.215 |
| Shared responsibility | 0.026 | 0.091 | 0.29 | 0.773 | -0.151 | 0.204 |
| Context of illness |  |  |  |  |  |  |
| Bi-polar | 0.150 | 0.057 | 2.62 | 0.009 | 0.038 | 0.262 |
| Heart disease | 0.507 | 0.057 | 8.87 | 0.000 | 0.395 | 0.619 |
| Context of scenario |  |  |  |  |  |  |
| Last record stated no intention to quit | -0.081 | 0.057 | -1.41 | 0.158 | -0.193 | 0.031 |
| Running behind schedule | 0.019 | 0.057 | 0.33 | 0.742 | -0.093 | 0.131 |
| Age |  |  |  |  |  |  |
| 25–34 (ref) | — | — | — | — | — | — |
| 25–44 | -0.207 | 0.099 | -2.08 | 0.037 | -0.402 | -0.012 |
| 35–44 | 0.001 | 0.088 | 0.01 | 0.992 | -0.172 | 0.174 |
| 45–54 | 0.138 | 0.143 | 0.96 | 0.336 | -0.143 | 0.418 |

Intention

| Predictor | Coefficient | Std. Error | z | p-value | 95% CI Lower | 95% CI Upper |
| --- | --- | --- | --- | --- | --- | --- |
| Frame |  |  |  |  |  |  |
| Professional obligation | 0.205 | 0.095 | 2.16 | 0.031 | 0.019 | 0.390 |
| Shared responsibility | 0.151 | 0.097 | 1.56 | 0.118 | -0.038 | 0.341 |
| Context of illness |  |  |  |  |  |  |
| Bi-polar | 0.209 | 0.065 | 3.20 | 0.001 | 0.081 | 0.337 |
| Heart disease | 0.799 | 0.065 | 12.23 | 0.000 | 0.671 | 0.928 |
| Context of scenario |  |  |  |  |  |  |
| Last record stated no intention to quit | -0.145 | 0.065 | -2.21 | 0.027 | -0.273 | -0.017 |
| Running behind schedule | -0.153 | 0.065 | -2.35 | 0.019 | -0.281 | -0.025 |
| Age |  |  |  |  |  |  |
| 25–34 (ref) | — | — | — | — | — | — |
| 25–44 | -0.055 | 0.106 | -0.51 | 0.607 | -0.262 | 0.153 |
| 35–44 | -0.041 | 0.094 | -0.44 | 0.661 | -0.226 | 0.143 |
| 45–54 | 0.264 | 0.153 | 1.73 | 0.084 | -0.035 | 0.564 |
| 55–64 | 0.943 | 0.283 | 3.34 | 0.001 | 0.389 | 1.497 |
| Over 65 | 0.882 | 0.476 | 1.85 | 0.064 | -0.051 | 1.814 |
| Gender |  |  |  |  |  |  |
| Male (ref) | — | — | — | — | — | — |
| Female | 0.173 | 0.083 | 2.08 | 0.038 | 0.010 | 0.336 |
| Non-binary / Other | -1.008 | 0.306 | -3.29 | 0.001 | -1.609 | -0.407 |
| Prefer not to say | 1.021 | 0.482 | 2.12 | 0.034 | 0.075 | 1.966 |
| Intercept | 3.617 | 0.109 | 33.04 | 0.000 | 3.403 | 3.832 |

**supplementary table s6**
mixed-effects models (unadjusted)

|  | **Desire** | | **Duty** | | **Intention** | |
| --- | --- | --- | --- | --- | --- | --- |
|  | **β** | **95% CI** | **β** | **95% CI** | **β** | **95% CI** |
| **Framing** |  |  |  |  |  |  |
| Professional Obligation | 0.10 | -0.06, 0.27 | 0.04 | -0.14, 0.22 | 0.16 | -0.03, 0.36 |
| Shared Responsibility | 0.11 | -0.05, 0.29 | 0.02 | -0.16, 0.20 | 0.13 | -0.07, 0.32 |
| **Context of Illness** |  |  |  |  |  |  |
| Bipolar Disorder | 0.20* | 0.08, 0.31 | 0.15* | 0.04, 0.26 | 0.21* | 0.08, 0.34 |
| Heart Disease | 0.56* | 0.44, 0.67 | 0.51* | 0.39, 0.62 | 0.80* | 0.67, 0.93 |
| **Context of Scenario** |  |  |  |  |  |  |
| No Intention to Quit | -0.07 | -0.18, 0.04 | -0.08 | -0.19, 0.03 | -0.14* | -0.27, -0.02 |
| Running Late | 0.05 | -0.06, 0.17 | 0.02 | -0.09, 0.13 | -0.15* | -0.28, -0.03 |

*p<0.05

S**upplementary table s7**
mixed-effects subgroup analysis by medical professional role status (adjusted for age and gender)

|  | **Desire** | | **Duty** | | **Intention** | |
| --- | --- | --- | --- | --- | --- | --- |
| GP | **β** | **95% CI** | **β** | **95% CI** | **β** | **95% CI** |
| **Framing** |  |  |  |  |  |  |
| Professional Obligation | 0.14 | -0.07, 0.35 | -0.07 | -0.30, 0.15 | 0.18 | -0.06, 0.42 |
| Shared Responsibility | 0.08 | -0.13, 0.29 | -0.02 | -0.26, 0.21 | 0.15 | -0.10, 0.39 |
| **Context of Illness** |  |  |  |  |  |  |
| Bipolar Disorder | 0.27* | 0.13, 0.42 | 0.23* | 0.08, 0.37 | 0.26* | 0.10, 0.42 |
| Heart Disease | 0.60* | 0.45, 0.75 | 0.49* | 0.35, 0.64 | 0.81* | 0.65, 0.97 |
| **Context of Scenario** |  |  |  |  |  |  |
| No Intention to Quit | -0.09 | -0.24, 0.05 | -0.02 | -0.17, 0.13 | -0.13 | -0.29, 0.02 |
| Running Late | 0.10 | -0.05, 0.24 | 0.05 | -0.10, 0.19 | -0.20* | -0.35, -0.04 |

*p<0.05

|  | **Desire** | | **Duty** | | **Intention** | |
| --- | --- | --- | --- | --- | --- | --- |
| Medical Student | **β** | **95% CI** | **β** | **95% CI** | **β** | **95% CI** |
| **Framing** |  |  |  |  |  |  |
| Professional Obligation | 0.05 | -0.17, 0.27 | 0.22 | -0.02, 0.46 | 0.25 | -0.01, 0.50 |
| Shared Responsibility | 0.14 | -0.08, 0.37 | 0.07 | -0.18, 0.32 | 0.17 | -0.09, 0.43 |
| **Context of Illness** |  |  |  |  |  |  |
| Bipolar Disorder | 0.06 | -0.12, 0.24 | 0.001 | -0.17, 0.17 | 0.12 | -0.09, 0.34 |
| Heart Disease | 0.47* | 0.29, 0.65 | 0.53* | 0.36, 0.69 | 0.80* | 0.58, 1.01 |
| **Context of Scenario** |  |  |  |  |  |  |
| No Intention to Quit | -0.05 | -0.23, 0.13 | -0.21* | -0.38, -0.04 | -0.18 | -0.39, 0.04 |
| Running Late | -0.03 | -0.21, 0.15 | -0.03 | -0.20, 0.14 | -0.08 | -0.29, 0.13 |

*p<0.05
